# The Role of INDY in Metabolic Regulation

**DOI:** 10.5936/csbj.201303020

**Published:** 2013-12-08

**Authors:** Diana M Willmes, Andreas L Birkenfeld

**Affiliations:** a Department of Endocrinology, Diabetes and Nutrition, Center for Cardiovascular Research, Charité – University School of Medicine, Berlin, Germany

**Keywords:** INDY, citrate, insulin resistance, diabetes, obesity, longevity

## Abstract

Reduced expression of the Indy (I'm Not Dead Yet) gene in D. melanogaster and C. elegans extends longevity. Indy and its mammalian homolog mINDY (Slc13a5, NaCT) are transporters of TCA cycle intermediates, mainly handling the uptake of citrate via the plasma membrane into the cytosol. Deletion of mINDY in mice leads to significant metabolic changes akin to caloric restriction, likely caused by reducing the effects of mINDY-imported citrate on fatty acid and cholesterol synthesis, glucose metabolism and ß-oxidation. This review will provide an overview on different mammalian SLC1 3 family members with a focus on mINDY (SLCl3A5) in glucose and energy metabolism and will highlight the role of mINDY as a putative therapeutic target for the treatment of obesity, non-alcoholic fatty liver disease and type 2 diabetes.

## Introduction

In *D. melanogaster* and *C. elegans* reduced expression of the non-electrogenic dicarboxylate and citrate transporter *Indy* (Acronym for **I**′m **N**ot **D**ead **Y**et) promotes longevity in a manner akin to caloric restriction, one of the most reliable interventions to prolong life span over a wide range of species [1, 2]. In mammals, *SLC13A5* encodes the Na^+^-coupled citrate transporter NaCT (we will use the alternative name mINDY throughout the review), which shares the highest sequence and functional similarity with *D. melanogaster* *Indy*.

*mINDY^-/-^* knockout mice are protected from diet induced obesity and insulin resistance that go along with excess caloric intake and aging [3]. The effect is mediated by a profound action of mINDY on mitochondrial metabolism in mice. Therefore, mINDY might serve as a therapeutic target for the treatment of obesity and type-2 diabetes. The purpose of this review is to summarize the role of mINDY in mammalian glucose and energy metabolism and describe the most recent advances on structure, expression, function and regulation of the protein.

## The SLC13A family – an overview

The SLC13A family of Na^+^-coupled di- and tri-carboxylate/sulfate transporters comprises five genes, namely *SLC13A1-5*, encoding multi-spanning transporters with 8-13 transmembrane α-helices flanked by an intracellular N-terminus and an extracellular C-terminus, containing putative consensus glycosylation sites [4]. Orthologs of all five genes are found in prokaryotes and eukaryotes. All SLC13A family members contain numerous predicted consensus phosphorylation and N-myristyoylation sites for which the functional significance is unknown so far. Moreover, a highly conserved consensus sequence motif, the “sodium:sulfate symporter family signature’’, is found in each of the five family members [5].

The mammalian SLC13A Na^+^-coupled anion cotransporters are located in the plasma membrane of epithelial cells with ubiquitous expression, but mainly in liver, kidney, small intestine, placenta and cells of the central nervous system. They all play a variety of different physiological and pathophysiological roles in the different organs. SLC13A transporters mediate the Na^+^-coupled anion substrate movement across the plasma membrane of cells and are electrogenic with a general Na^+^:substrate ratio of 3:1.

SLC13A family members are divided in two functional groups: the Na^+^-sulfate cotransporters (NaS) mainly transporting sulfate, selenate and thiosulfate and the Na^+^- di- and tri-carboxylate cotransporters (NaDC) carrying Krebs-cycle intermediates such as succinate, citrate and α-ketoglutarate. While the *SLC13A1* and *SLC13A4* genes belong to the NaS, the *SLC13A2*, *SLC13A3* and *SLC13A5* genes represent the NaDC group.

SLC13A1 (also NaS1 or NaSi-1) is localized to the apical brush border membrane of the renal proximal tubules and intestinal epithelial cells [6–9]. SLC13A1 functions as an electrogenic pH-sensitive high affinity Na^+^-dependent SO^-2^_4_ transporter with substrate preferences for the anions sulfate, thiosulfate, selenate and the cation Na^+^ [10, 11]. The human SLC13A1 transporter can be inhibited by molybdate, selenate, tungstate, selenate, succinate and citrate [6]. SLC13A1 deficient mice revealed numerous pathophysiological features, such as hyposulfatemia, hypersulfaturia, reduced body weight, postnatal growth and fertility, reduced circulating steroid levels, increased urinary glucocorticoid excretion and altered lipid and cholesterol metabolism within the liver [12–19]. The loss of SLC13A1 clearly shows its importance in maintaining sulfate homeostasis.

SLC13A2 is localized in epithelia with high metabolic needs specifically on the apical membrane of renal proximal tubular and small intestine cells where it reabsorbs intermediates of the tricarboxylic acid cycle like succinate, α-ketoglutarate and citrate. The activity and cell surface expression of SLC13A2 is dependent on its regulation by PKC-dependent, direct-phosphorylation independent pathways including the serum and glucocorticoid inducible kinases SGK1 and 3, PKB kinase and the NHE regulating factor 2 (NHERF2) [20, 21]. By taking up tricarboxylic acid (TCA) cycle intermediates into cells across the apical membrane, SLC13A2 plays an important role in oxidative metabolism. *SLC13A2^-/-^* mice are characterized by increased urinary excretion of diverse dicarboxylates [22].

The main function of SLC13A2 is renal handling of citrate and therefore important in the formation of kidney stones and nephrolithiasis [20, 23]. Since the SGK1 signaling pathway, contributing to the regulation of renal function and arterial blood pressure, activates SLC13A2, the protein may also be important in the regulation of blood pressure, in addition to its role in water re-absorption [24].

SLC13A3 is conserved over a wide range of species and has been detected in zebrafish, xenophus frog, mouse and human [25]. SLC13A3 is expressed in liver, brain, kidney, placenta, pancreas, eye and optic nerve and is located on the apical membrane of placenta and synaptosomes and on the basolateral membrane of hepatocytes and renal proximal tubular cells. Slc13a3 is found mainly in astrocytes and at lower degree in neurons within the central nervous system [26–31]. SLC13A3 shows a substrate preference for succinate, α-ketoglutarate and citrate and is inhibited by TCA cycle intermediates, such as fumarate, oxaloacetate or malate [32]. So far, no SLC13A3 deficient mouse model has been described, but it was reported that renal mRNA and protein expression levels increase with age in humans and rats [33]. Moreover, SLC13A3 activity and plasma membrane expression are regulated via PKC-dependent and -independent mechanisms [34, 35]. Moreover, SLC13A3 seems to be involved in the regulation of cellular senescence by a mechanism including the NAD-(+)-dependent histone deacetylase sirtuin-1 (SIRT1) [36]. *SLC13A3* expression has been shown to be regulated by the transcription factor paired-like homeodomain transcription factor 2 (PITX2) in ocular cells and was suggested to be important in the inborn metabolic disease Axenfeld-Rieger syndrome type 1 (ARS1), an autosomal dominant disorder of morphogenesis. ARS1 is caused by mutations in *PITX2* leading to abnormal eye anterior segment development which could result in glaucomatous blindness [37]. Moreover *SLC13A3* was identified by genomic analysis to be associated with hypertension and type-2 diabetes [38–40].

SLC13A4 is mainly expressed in placenta and testis and at a lower extent in brain, heart, thymus, liver and tonsils [41, 42]. The Na^+^-dependent transport of sulfate can be inhibited by substrates with similar structures like molybdate and selenate most likely by substrate competition [42]. So far no SLC13A4 null mouse model has been described, and remains to be determined whether or not SLC13A4 is involved in any human disorder (for an overview see Table 1).

**Table 1:** Relevant data on SLC13A family members.

Gene	Main expression	Substrates	Physiological Relevance
Slc13a1	Renal proximal tubulus	Sulfate, Thiosulfate	sulfate homeostasis
	Intestinal epithelial cells	Selenite	
		Na^+^	
Slc13a2	Metabolically relevant epithelia	Succinate	Tri/Dicarboxylate homeostasis
	Renal proximal tubulus	α-Ketoglutarate	
	Intestinal epithelial cells	Citrate	
Slc13a3	Brain	Succinate	Tri/Dicarboxylate homeostasis cellular senescence (?),
	Kidney	α-Ketoglutarate	Axenfeld-Rieger Syndrome type I
	Placenta	Citrate	
	Liver Pancreas		
	Eye optic nerve		
Slc13a4	Placenta	Sulfate	Unknown
	Testis		
	Brain Heart		
	Thymus		
	Liver		
	Tonsils		
Slc13a5	Liver	Citrate	Tri/Dicarboxylate homeostasis metabolic processes such as fatty acid synthesis, gluconeogenesis
	Testis	Succinate	
	Brain	Malate Fumarate	

## SLC13A5 – structure, expression and function

**Figure 1:**
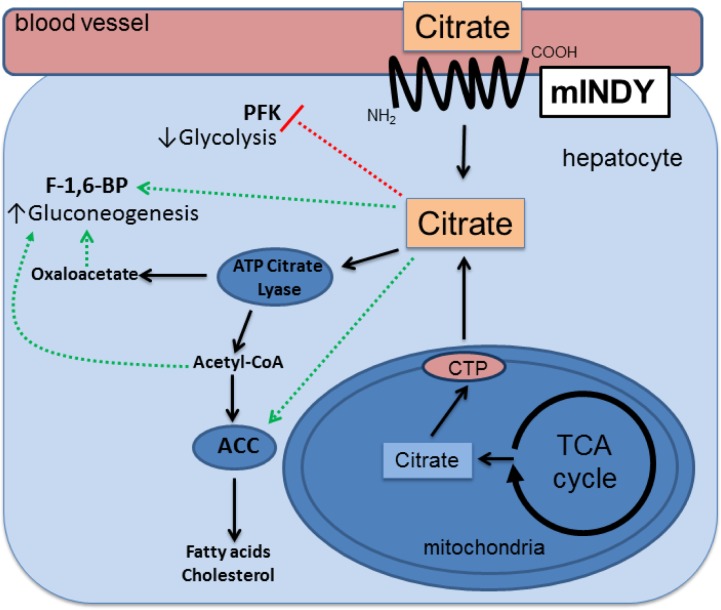
Schematic representation of metabolic pathways affected by intracellular citrate molecules.

Cytosolic citrate is known as the prime carbon source for the synthesis of fatty acids, triacylglycerols, cholersterols and low-density lipoproteins. Moreover citrate leads to the activation of fatty acid synthesis and affects glycolysis and ß-oxidation [43–45]. An overview is provided in Figure 1. Main organs for fatty acid synthesis are the liver and white adipose tissue. Fatty acid synthesis has been shown to directly correlate with cytosolic citrate concentrations, partially depending on the direct import across the plasma membrane by mINDY [46, 47].

Initially, the significance of *Indy* in lifespan and metabolic regulation has been described in *D. melanogaster*, where it encodes an electroneutral tricarboxylate carrier, mainly expressed in organs involved in energy homeostasis and preferentially transporting citrate across the plasma membrane in flies [2, 46, 48–54]. Long lived flies with reduced expression of *Indy* showed a reduction in whole body fat stores and less expression of insulin like proteins, comparable to levels in calorically restricted wildtype flies [55].

The mammalian Indy homolog, mINDY, or SLC13A5, was identified as the latest member of the SLC13 gene family and consists of 11 transmembrane domains [46]. The bacterial INDY homologue protein binds one molecule of citrate and one molecule of sodium. Here, the binding sides exist of conserved amino acid proteins, serving as the structural basis of the transporter specificity, which is conserved over a wide range of species [48]. The Na^+^:citrate stoichiometry for mINDY is 4:1, which is in contrast to the other SLC13 family members. The *SLC13A5* gene encodes the Na^+^-coupled citrate transporter mINDY (NaCT) and shares the highest sequence and functional similarity with *D. melanogaster* Indy. In humans, the *INDY* gene is located on chromosome 17p13 with a size of approximately 30kb consisting of 12 exons [46]. A splice variant lacking 43 N-terminal residues has been reported by Pajor and colleagues, the function and tissue distribution are still unknown [56]. Mammalian tissue distribution studies have localized predominant INDY expression within the liver, but also in testis and brain[3, 46, 51]. Within neuronal tissues, *mINDY* shows an exclusive expression pattern in neurons but not in astrocytes [27, 29].

mINDY has a vast substrate specificity for the tricarboxylate citrate and exhibits the inward electrogenic Na^+^-coupled substrate cotransport. It has been reported, that mINDY mediated citrate transport is pH-sensitive and maximal activity is exhibited at pH 7.0-7.5, whereas the transporter is inhibited in acidic and alkaline pH [52].

The transporter shows lower affinities to other Krebs-cycle intermediates such as succinate, malate or fumarate [46, 51, 52]. mINDY is less selective to other organic anions since it is not mediating the uptake of glutarate derivates into neurons [57]. Moreover, mINDY is Li^+^-sensitive, varying largely between species. While human mINDY is stimulated by Li^+^, it is inhibited or unaffected in other species [47, 58].

Several lines of evidence suggest that transcriptional regulation of *mINDY* is mediated by the nutritional state, suggesting metabolic factors being involved in the regulation. For example, *Indy* expression is reduced by caloric restriction in flies [55], whereas caloric restriction increases *INDY* expression in *bicyclus anynana* butterflies [59]. In mice, starvation over 36 hours reduces *mINDY* expression in the liver [3]. In contrast, gavaging large amounts of olive oil induced *INDY* expression strongly in the liver in rats, as identified by microarray assays [60]. Etcheverry and colleagues could show that the regulation of *mINDY* is mediated by epigenetic mechanisms. Profiling of whole genome integrative analysis of methylation and gene expression in glioblastoma patients possessed several genes, showing an inverse correlation between promoter methylation and expression level in glioblastomas. Moreover, *INDY* was down-regulated with hypermethylation of its promoter [61].

Recently, we have demonstrated that mINDY expression and citrate uptake was induced by physiological concentrations of the hormone glucagon via a cAMP and cAMP-responsive-element-binding-protein (CREB)- dependent mechanism. The promoter sequence of mINDY was identified including a CREB binding site within this fragment, identifying mINDY as a CREB-dependent glucagon target gene, which is induced in the short term fasting status and type-2 diabetes [62].

Since mINDY is predominately expressed in the liver, a metabolically highly active organ, it may play a role in various metabolic processes in which citrate has an important function such as mitochondrial energy production (TCA cycle), fatty acid synthesis, cholesterol synthesis, glycolysis and gluconeogenesis [58].

Citrate is imported from the circulation across the plasma membrane into the liver by an electrogenic citrate-sodium co-transport via mINDY (NaCT) and other family members of the SLC13 transporter family. Citrate generated within the mitochondria by each turn of the TCA cycle also enters the cytoplasm when intracellular energy stores are abundant. Cytoplasmic citrate is converted to acetyl-CoA by ATP-citrate lyase (ACLY). The conversion of acetyl-CoA to malonyl-CoA by acetyl-CoA carboxylase (ACC) is the first step in fatty acid synthesis. ACC gets allosterically activated by citrate. The product from this reaction, malonyl-CoA, serves as the donor of C2-acetly groups in each turn of the fatty acid synthesis reaction cycle. The ACLY reaction also yields oxaloacetate, which can be decarboxylated and phosphorylated to form phosphoenolpyruvate (PEP), through phosphoenolpyruvate carboxykinase. A (PEPCK), opening into gluconeogenesis. Moreover, acetyl-CoA can be routed into cholesterol synthesis via several enzymes including 3-hydroxy-3-methylglutaryl-coenzym-A-reductase (HMG-CoA-reductase).Green lines indicate activating and red lines inhibitory connections. ATP – Adenosine triphosphate, CTP – citrate transport protein, F-1,6-BP – Froctose-1,6-bisphosphatase, PFK – Phosphofructokinase, TCA – tricarboxylic acid.

## Regulation of citrate homeostasis by SLC13 members

Serum citrate concentration is relatively constant, ranging from 50-200 µM [62, 63]. Citrate absorption from nutritional sources in the small intestine seems to be mediated mainly via NaDC1 and NaDC3 [64, 65]. By this mechanism, more than 90% of an oral citrate load can be absorbed and hypocitraturia can be ameliorated [64, 66]. At physiological pH, most of serum citrate circulates in the form of triply charged citrate bound to divalent ions, such as calcium and magnesium, and is filtered freely at the glomerulus; reabsorption takes place predominantly in the proximal tubule via NaDC1 [65]. Moreover, citrate is also taken up into the kidney by removal from postglomerular blood. Interestingly, similar to our findings in the liver, citrate is also oxidized in the TCA cycle in the kidney and it has been reported to be metabolized to glucose in gluconeogenesis in the kidney [3, 62]. By these mechanisms, citrate contributes to meet the energy needs of the kidney [67–70]. In addition to kidney, citrate is also taken up by the liver via mINDY and NaDC3. There, it can be oxidized or it can be used for the synthesis of fatty acids and glucose (Figure 1) [62].

Circulating citrate is released from muscle, skin and bone. Citrate concentrations in bone exceed that of most other tissues and accounts for 70% of total body citrate content^71^. Citrate can also be taken up into the bone, being deposited in the mineral fraction and mINDY has recently identified in bone matrix, probably contributing to the function [71, 72]. Skeletal muscle, with its high capacity for oxidative substrate utilization is probably a major source of citrate release to the plasma pool. The isolated rat hindquarter releases considerable amounts of citrate [73]. Arterio-femoral plasma citrate difference in the working man is largely negative, and the working muscle can be considered as one of the major sources of plasma citrate during exercise [74]. Moreover, the human heart has also been shown to release citrate, and is has been postulated that similarly to liver and kidney, this function may contribute the regulation of myocardial lipid and glucose metabolism [75].

The physiological significance of the regulation of plasma citrate homeostasis is largely unknown. The plasma halflife of citrate in the dog is about 20 minutes, and citrate turnover rates have been estimated to equal 250µmol/h/L in man, with a tissue to plasma gradient of 3-4 to 1 [70]. Externally administered citrate is oxidized to a major extend and it might, hence, contribute to furnish cellular energy needs. Citrate concentrations undergo significant circadian rhythmicity peaking in the postprandial phase, a pattern that seems to be disturbed in type 1 diabetics [76, 77]. In these patients, insulin decreases circulating citrate levels. Prolonged fasting also reduces citrate levels in men. Moreover, we have found that glucagon increases the expression of SLC13A5 via the transcription factor CREB in rats, and by this mechanism, increases the uptake of citrate into the liver in early fasting [62].

## Metabolic regulation through SLC13A5

INDY might be involved in metabolic regulation since it transports intermediates of the TCA cycle and reducing the expression of *Indy* promotes longevity in a manner akin to caloric restriction in flies [55, 78]. Caloric restriction was shown to prolong life span, or at least health span, in many species including primates [79–83].

Deletion of the *Indy* homolog *ceNAC-2* in *C.elegans* leads to prolonged lifespan with a reduction in whole body fat stores [2, 53, 55, 84]. In *D. melanogaster*, *Indy* encodes a non-electrogenic, dicarboxylate and citrate transporter mainly expressed in organs of intermediary metabolism, i.e. the fat body, midgut and oenocyte [2]. The mammalian protein *mINDY* shares highest sequence and functional similarity with the fly *Indy* gene [46].

To examine the metabolic effects mINDY might have in mammals, we created a whole body knockout mouse model (*mINDY^-/-^* mouse). *mINDY^-/-^* mice displayed a reduction in the uptake of citrate from the circulation into the liver, but not kidney and adipose tissue, paralleled by elevated circulating citrate levels [3, 85]. *mINDY^-/-^* mice did not gain as much weight as control mice, a phenotype getting more pronounced with age. The observation was accompanied by a somewhat unexpected increase in oxygen consumption, carbon dioxide generation and resting energy expenditure. In this vein, hepatic mitochondrial density and gene expression of the peroxisome proliferator-activated receptor-gamma coactivator 1 alpha (PGC1-α) were increased in *mINDY^-/-^* mice. Hepatic microarray studies revealed important similarities between *mINDY^-/-^* mice and calorically restricted mice, sharing ∼ 80% of similar transcriptional changes, including increased expression of electron transport chain components.

Consequently, on a high fat diet, *mINDY^-/-^* mice showed less body weight gain compared to control mice. Moreover, fat mass was significantly reduced by almost 50% and relative lean mass was increased as measured by nuclear magnet resonance. Hepatic triglyceride content was reduced upon mINDY deficiency concomitant with reduced liver lipid deposits. Moreover, the hepatic lipid oxidation marker, beta-hydroxybutyrate, was increased in high caloric fed mice by 62%. Measurements of lipid oxidation in primary hepatocytes isolated from high caloric diet fed *mINDY^-/-^* mice revealed a reduced incorporation of citrate into fatty acids and sterols. Together, these data indicate that high fat diet fed mINDY knockout mice have elevated lipid oxidation and reduced lipid synthesis rates compared to controls [3].

AMP-activated protein kinase (AMPK) is activated by increased intracellular adenosine monophosphate (AMP) stores and inhibited when adenosine triphosphate (ATP) reservoirs are high. Hence, AMPK is able to serve as a cellular energy sensor, coordinating insulin sensitivity, lipid-oxidation and -synthesis and mitochondrial biogenesis via the transcriptional co-activator *PGC1-α* [86, 87] In the mINDY^-/-^ mice, ATP stores were reduced and AMPK phosphorylation was increased, suggesting an increased activation of AMPK through reduced intracellular biochemical energy stores [3].

Feeding mice a high fat diet serves as a well-known model to study obesity related impairment in glucose metabolism [88–92]. For this purpose, mINDY^-/-^ mice were fed a high fat diet, and the impact of mINDY deletion on glucose metabolism was studied *in vivo*, by intraperitoneal glucose tolerance tests (ipGTT) first. Basal plasma glucose and insulin concentrations were decreased in mINDY^-/-^ mice compared to control mice. Then, the gold standard to study insulin sensitivity, the hyperinsulinemic euglycemic clamp, demonstrated improved insulin sensitivity with reduced basal and clamp endogenous hepatic glucose production upon mINDY deletion. Additionally, peripheral glucose uptake by the gastrocnemius muscle was increased in INDY deletion mice. This protection from fat-induced muscle insulin resistance was accompanied by reduced content of skeletal muscle DAGs as major mediators of insulin resistance [93–97].

Hepatic insulin resistance is strongly associated with hepatic lipid accumulation [98]. A simple unifying hypothesis that has been proposed to explain these observations is that insulin resistance develops when there is an imbalance between supply and utilization of intracellular lipid leading to net accumulation of intracellular diacylglycerol (DAG hypothesis) [93, 98, 99]. Increased DAG content in turn results in activation of novel protein kinase C's (PKC's) and subsequent impairment in insulin signaling. With high-fat feeding, this condition is achieved due to the continued, high supply of dietary fat, which exceeds the capacity of hepatocytes to oxidize the fatty acids, store the fatty acids as neutral lipid or export fatty acids in VLDL (very low-density lipoprotein) particles. In contrast, with aging, declines in mitochondrial function may contribute to net accumulation of intracellular DAGs [99]. Consistent with this hypothesis, *mINDY^-/-^* mice showed reduced hepatic DAG concentrations, decreased membrane PKCɛ content, and protection from hepatic insulin resistance associated with high-fat feeding and aging. Confirmation of this key interaction between DAG, activation of PKCɛ, and insulin resistance has been demonstrated in numerous other rodent models of fatty liver associated hepatic insulin resistance [97, 100–106].

## SLC13A5 – therapeutic aspects

Reducing *INDY* expression has been proven beneficial in terms of life span and/or metabolic regulation in all species tested so far. Therefore, it seems plausible to speculate that mINDY might be an interesting target for the treatment of metabolic disease, such as obesity, non-alcoholic fatty liver disease and type 2 diabetes [48, 107, 108]. To date, no specific therapeutic agents to modulate mINDY function or expression have been reported. Interestingly, human mINDY activity was described to be stimulated by Lithium, in concentrations that are observed during the treatment of bipolar disorders. Sun and colleagues reported on compounds, which inhibit the citrate transport protein (CTP) on the inner mitochondrial membrane [109, 110]. Moreover they could identify a compound with selectivity for mINDY over CTP [111]. Whether or not such a compound will be amenable to therapeutic intervention remains to be determined. The high concentration needed to inhibit mINDY with this molecule makes it unlikely to become clinically relevant. The discovery of a more potent compound modulating mINDY function could provide a useful tool to delineate the structure and function of mINDY. Ultimately, a putative inhibitor of mINDY holds the potential to induce the beneficial effects of caloric restriction, without requiring severe caloric restriction in mammals.
